# Transcriptomic and Physiological Analysis Reveals the Possible Mechanism of Inhibiting Strawberry Aroma Changes by Ultrasound after Harvest

**DOI:** 10.3390/foods13142231

**Published:** 2024-07-16

**Authors:** Yutong Li, Siyue Liu, Huiyu Kuang, Junyi Zhang, Bei Wang, Shaojia Wang

**Affiliations:** Beijing Advanced Innovation Center for Food Nutrition and Human Health (BTBU), School of Food and Health, Beijing Higher Institution Engineering Research Center of Food Additives and Ingredients, Beijing Technology and Business University, Beijing 100048, China; 2130021015@st.btbu.edu.cn (Y.L.); friedb62y@163.com (S.L.); khy1128@126.com (H.K.); zjy2030202087@163.com (J.Z.); wangbei@th.btbu.edu.cn (B.W.)

**Keywords:** strawberry, ultrasound, flavor, transcriptomic, volatile organic components, GC-MS

## Abstract

The volatile compounds in strawberries play a significant role in the formation of strawberry aroma. However, these compounds undergo continual changes during storage, resulting in a decline in quality. In this study, a total of 67 volatile organic compounds (VOCs) were identified in strawberries through quantitative analysis. At the end of the storage period, the VOC content in the ultrasonic group was 119.02 µg/kg higher than that in the control group. The results demonstrated that the ultrasonic treatment increased the contents of terpenes and esters at the end of storage. Among these, linalool increased from 67.09 to 91.41 µg/kg, while ethyl cinnamate increased from 92.22 to 106.79 µg/kg. Additionally, the expression of the key metabolic genes closely related to these substances was significantly up-regulated. The expression of the *FaNES* gene, related to terpene metabolism, was up-regulated by 2.8 times in the second day, while the expression of the *FaAAT* gene, related to ester metabolism, was up-regulated by 1.5 times. In summary, this study provides a theoretical basis for exploring the mechanism of ultrasonic effect on strawberry flavor and quality after harvest.

## 1. Introduction

Strawberry (Fragaria × ananassa Duch) is an octaploid (2*n* = 56) perennial root herb of the rose family and is widely known as the “queen of fruits”. It is characterized by a bright red appearance and a distinctive fruit shape, with a sweet taste and a tender and juicy texture [1]. Strawberries are rich in amino acids, vitamins, flavonoids and other nutrients, with a high level of consumer acceptance [2]. However, strawberries are prone to damage due to their high water content and absence of a hard peel, making them softened and infested with mold and other microorganisms, leading to quality deterioration after harvest [3,4,5]. Ultrasonic treatment can extend the shelf life of fruits by inhibiting the growth of harmful microorganisms and regulating the enzyme activity of key metabolic pathways. Feng et al. demonstrated that ultrasonic treatment could enhance the antioxidant capacity of grapes and improve the quality of fruits [6]. Previous studies have also proved that ultrasonic treatment could affect the transcriptome gene expression of strawberries and alleviate fruit softening [7].

The scientific name of strawberry, ‘Fragaria’, refers to the aromatic aspect, which is one of the important characteristics of fresh strawberries [8]. Typically, aroma is produced by volatile organic compounds (VOCs) and can be used as an indicator of fruit quality deterioration [9,10]. Although the total content of the VOCs only accounts for 0.001% to 0.01% of the fresh fruit mass, they exert a significant impact on the aroma and flavor of strawberries [3]. Previous studies have identified over 350 volatile substances in strawberries, with the majority belonging to the classes of esters, ketones, alcohols, terpenes, aldehydes, and acids [11,12]. The aroma of fruit is not only influenced by the type and content of VOCs, but also by their threshold. According to the aroma activity value OAV (content/threshold), VOCs can be classified into characteristic aroma components or non-characteristic aroma components [13]. Characteristic aroma components are aroma components representing the characteristic aroma of the fruit. As for strawberries, the characteristic aroma components include terpenoids, aldehydes, esters, and furanones. Terpenes are important secondary metabolites in plants and serve as the primary components affecting the floral flavor of many flowering plants. Of them, linalool and nerolidol have a greater effect on the aroma of strawberries and are responsible for the production of floral and citrus fragrances [14]. Aldehydes impart herbal aromas, with hexanal and hexenal being the most common in fruits [15,16].

The content of ester compounds in ripe strawberry fruits accounts for 25% to 90% of the total VOCs, which is an important part of the characteristic aromatic substances of strawberries [17,18]. The ester substances in strawberries mainly include methyl esters and ethyl esters. Among these, methyl butyrate, ethyl butyrate, methyl caproate, and ethyl caproate have been identified as the principal aromatic compounds of strawberry fruit, imparting a sweet, fruity aroma to the fruit [19]. Although the content of furanone in strawberries is relatively low, 4-Hydroxy-2,5-dimethyfuran-3(2H)-one (DMHF) and 4-Methoxy-2,5-dimethyfuran-3(2H)-one (DMMF) are referred as the most significant aromatic components. These components exert a significant impact on the aroma of strawberries, imparting caramel, fruit, and burnt pineapple aromas [20,21].

During storage, strawberries continue to metabolize, followed by the synthesis and metabolism of aroma compounds [15]. Previous studies have shown that both the type and content of the esters in strawberries increase during storage [3], especially ethyl acetate, imparting a distinctive fruity flavor. This is mainly related to the metabolism of fatty acids and amino acids in strawberry species [22,23,24]. Furthermore, some studies have found that storage can lead to a decrease in the alcohol content, but some specific alcohols, such as 1-propanol and 3-octanol, induce quality deterioration with their increased content during storage [22,25]. The consumption of other substances such as sugar [26], leading to the continued synthesis and increased contents of furanone metabolites [27,28]. In addition to the influence of storage time on aroma, many preservation treatments such as chilling, irradiation, different types of packaging, etc., have also been reported to have a significant influence on fruit aroma [29,30].

Ultrasonic treatment is an efficient method for maintaining the freshness of fruits. Additionally, it has been reported to alleviate the quality deterioration of fruits during storage [31,32]. Ultrasonic technology differs from other preservation technologies and is often used in auxiliary processing [33,34,35]. Previous studies have shown the effects of ultrasound on processing [36], cleaning [37], and sterilization [38]. Nevertheless, reports on the impact of ultrasound on the aroma of fruits, particularly strawberries, are scarce. Meanwhile, there are few studies on the influence of ultrasound on the transcriptome of fruits during storage. Consequently, this study analyzed the changes in transcriptomics and VOCs to investigate the impact of ultrasonic waves on the aroma metabolism of strawberries during storage. Overall, the experimental findings will provide a theoretical and experimental basis for future studies on the aroma changes of fruits and vegetables during storage and offer insights for the practical application of ultrasonic wave preservation.

## 2. Materials and Methods

### 2.1. Experimental Material

The octaploid “Red face” strawberry (Fragaria × ananassa Duch. cv. “Benihoppe”) from Da Zi Ran farm (39.54° N, 116.23° E, Beijing, China) was selected as the experimental material. Strawberry plants were grown in a greenhouse containing a mixture of nutrient soil, vermiculite, and organic fertilizer (7:2:1, *v*/*v*/*v*). The seedlings were spaced 0.2 m within each row column and grown in a controlled environment with the following conditions: 25 °C:18 °C (day:night), 60% humidity, 12 h photoperiod. The samples were obtained by organic planting methods, and organic fertilizer made of fish protein was used. And insect infestations were prevented by physical techniques such as insect nets. Fresh strawberry fruits of similar ripening quality were transported to the laboratory immediately after picking. Strawberry fruits with similar size, color, maturity, and no obvious surface damage were pre-cooled at 5 °C for 24 h for use. The strawberries were randomly divided into a control group (CK) and an ultrasonic group (U) for treatment. Ultrasonic group (U): the strawberries were completely immersed in an ultrasonic tank filled with deionized water for ultrasonic treatment. The ultrasonic conditions were the same as in the previous study [7]: ultrasonic frequency was 28 kHz, ultrasonic power was 0.15 W/cm^2^, and ultrasonic time was 3 min (to maintain a constant water temperature in the ultrasonic tank, the ultrasonic equipment (Beijing Jinxing Ultrasonic Equipment Technology Co., Ltd., Beijing, China) was connected to a low-temperature constant water bath, and the temperature was set at 15 °C). Control group (CK): the strawberries were completely immersed in deionized water for the same time as the ultrasonic group. After being treated, the surface water of the strawberries was removed with absorbent paper and then they were stored in a refrigerator at 5 °C and a humidity of 75–85%.

### 2.2. Determination of Volatile Compounds

Volatile compounds were collected by headspace solid-phase microextraction [39,40]. The strawberry samples from the ultrasonic group and the control group were randomly selected at 0 h, 3 h, 6 h, 9 h, 12 h, 1 d, 2 d, 3 d, 6 d, 9 d, 12 d, and 15 d during storage. After removing the leaf stalks, the strawberries were cut into parts weighing 5 g each and mixed with 5 mL saturated NaCl (>99%) solution. Then, 3 g of fruit pulp was placed in a 15 mL headspace bottle and the internal standard 2-methyl-3-heptanone (>99%) (0.816 mg/mL, 1 μL) was added. After the sealed headspace bottle was temperated in a water bath at 25 °C for 20 min, the SPME arrow extraction head was inserted into the headspace bottle and the extracted fiber was pushed out. After 30 min of headspace adsorption, the extracted fiber was retracted and the extraction head was pulled out. Each group was treated in triplicate per time point.

The volatile compounds were determined by gas chromatography–mass spectrometry (Agilent Technologies, Santa Clara, CA, USA) following the previously reported method with some modifications [41]. GC condition: DB-WAX column, 60 m × 0.25 mm × 0.25 μm, the carrier gas was helium at a flow rate of 1.2 mL/min. The temperature procedure of the column was as follows: the initial temperature of the column was 40 °C, then temperature increased to 75 °C at 7 °C/min; it was heated up to 150 °C at 2 °C/min; and the temperature was raised to 230 °C at 5 °C/min for 2 min. The diversion mode was adopted. MS conditions: electron ionization source, electron energy 70 eV, the inlet temperature was 250 °C, the ion source temperature was 230 °C, the quadrupole temperature was 150 °C, full scanning mode, and the quality scanning range was *m*/*z* 35~350.

### 2.3. Transcriptome Sequencing

The strawberry samples from the ultrasonic group and the control group were randomly selected at 0 h, 3 h, 6 h, 9 h, 12 h, 1 d, 2 d, 3 d, 6 d, and 9 d during storage. The strawberries were immediately beaten and mixed (5 s) after the leaf stem was removed and divided. About 10 g of strawberry pulp was weighed and packed in a 10 mL centrifuge tube, frozen with liquid nitrogen, and then refrigerated at −80 °C. For transcriptome sequencing, each treatment group was set up with 3 biological replicates per time point. The sequencing of strawberry samples, including total RNA extraction and quality inspection, cDNA library construction, transcriptome sequencing, and preliminary bioinformatics analysis, was performed by the Shanghai Meiji Biomedical Technology Co., Ltd. (Shanghai, China). The gene differential expression analysis was conducted using DESeg2 1.24.0 software [42]. The screening criteria for differential expression genes were |log_2_FC| > 1 (difference multiple > 2) and *p*-adjust < 0.05.

All genes were functionally annotated in the GO, KEGG, COG, NR, Swiss-Prot, and Pfam databases. The gene function enrichment analysis mainly includes KEGG enrichment analysis to identify the genes involved in the metabolic pathways. The KEGG enrichment analysis was performed by Goatools 0.6.5 software, and the *p*-value was corrected by default. The KEGG pathway with *p*-adjust < 0.05 was significantly enriched in gene concentration.

### 2.4. Real-Time Fluorescence Quantitative PCR

The key genes related to the signal pathway of strawberry active aroma substances were selected and the cDNA samples of the ultrasonic group and the control group were used for real-time fluorescence quantitative PCR (qPCR) to verify the reliability of the transcriptome sequencing results. The octoploid strawberry Actin gene was used as the internal reference gene. The selected gene coding region used Primer Premier 5.0 software design primers through the Primer-BLAST (https://www.ncbi.nlm.nih.gov/tools/primer-blast (accessed on 15 January 2022)) for their early specific validation. The specificity of the primers was confirmed by the dissolution curve. The internal reference Actin primer was F: 5′-GGTGACGAGGCTCAATCCAA-3′, R: 5′-GGGCAACACGAAGCTCATTG-3′, and the primer sequence of the target gene is shown in Table 1.

After the total RNA was qualified, cDNA with a concentration of 50 ng/uL was synthesized by reverse transcription and freeze-dried at −20 °C for further use. Later, the sample was diluted 10 times for use. The gene-specific primers were synthesized by Suzhou Hongxun Biotechnology Co., Ltd. (Suzhou, China), and real-time fluorescent quantitative PCR experiments were performed using the SuperReal Premix Plus (SYBR Green) kit (Tiangen Biochemical Technology Co., Ltd. Beijing, China). Real-time fluorescence quantitative PCR reaction system and the procedures were as follows: a 10 μL real-time fluorescence quantitative PCR reaction system: 2 × SuperReal Premix Plus (SYBR Green) 5 μL; Template cDNA 5 ng; 5 μM Primer F, 0.3 μL; 5 μM Primer R, 0.3 μL; ddH_2_O was added to 10 μL. After adding a 10 μL real-time fluorescence quantitative PCR reaction system, the real-time fluorescence quantitative PCR reaction was performed using the CFX96 real-time fluorescence quantitative PCR instrument (BioRad, Hercules, CA, USA). The 10 μL real-time fluorescent quantitative PCR reaction procedure was as follows: predeformation stage 95 °C, 15 min; cycle reaction stage 95 °C 10 s, 55 °C 20 s, 72 °C 20 s, a total of 40 cycles; dissolution curve stage 65 °C 5 s, 95 °C 15 s.

Based on the expression level of each gene in the CK-0H samples, 2^−ΔΔCT^ was used to calculate the relative expression level of the target genes [43]. Each gene in each sample was performed with 3 duplicate holes, and template-free amplification was used as a negative control.

### 2.5. Data Processing

The data significance and correlation analyses were performed using IBM SPSS Statistics 27. The data were analyzed and mapped using Excel 2021 and Origin 2022 software. Transcriptome-related data were analyzed and mapped using various online data processing and mapping tools provided by the Meggie Biocloud platform (http://www.majorbio.com/ (accessed on 19 January 2024)).

## 3. Results and Discussion

### 3.1. Analysis of Volatile Organic Compound Types of Strawberry during Storage

**Figure 1:**
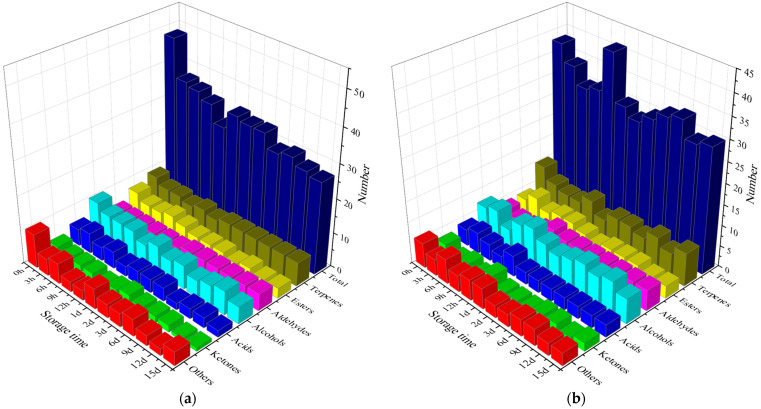
Number of volatile organic components in strawberry fruit during storage. (**a**) Control group; (**b**) ultrasonic group.

The types of volatile compounds in strawberries during storage mainly include esters, terpenes, alcohols, and aldehydes. As shown in Figure 1 and Table A1, ultrasonic treatment alleviated the reduction of VOC types during the storage period. A total of 67 volatile compounds were detected in strawberries by gas chromatography–mass spectrometry (attached table). Among them, esters accounted for the largest number (13 types), followed by terpenes (10 types), aldehydes (7 types), alcohols (11 types), acids (7 types), ketones (5 types), and other substances (14 types). A total of 53 aroma substances were identified in the ultrasonic group, including 10 terpenes, 10 alcohols, nine esters, four ketones, six aldehydes, five acids, and nine other substances. A total of 64 aroma substances were identified in the control group, including 10 terpenes, 11 alcohols, 12 esters, four ketones, six aldehydes, seven acids, and 14 other substances. This result was consistent with the findings of Ana et al. and Ulrich et al., reporting that strawberry aroma is mainly composed of esters, terpenes, and alcohols [40,44].

As shown in Figure 1, a total of 48 and 38 volatile compounds were detected in the control and ultrasonic groups at the beginning of the storage period. This might be because the initial abiotic stress, such as ultrasound, inhibits the metabolism of VOCs [45]. Strawberries have a reduced range of volatiles during storage, similar to some fruits stored in cold stores [46]. Compared with the sample at 0 h, the types of volatile compounds in both groups decreased at 15 d, and the types of volatile compounds in the ultrasonic group were more than three compared to those in the control group at 15 d. These results indicated that ultrasound inhibited the reduction in volatile species during storage. Cai et al. and Caleb et al. found that preservation treatments such as heat treatment and air conditioning could also mitigate this trend [45,47].

Among the volatile compounds, there were more types of terpenoids and alcohols, and the number of alcohols in the control group decreased with the extension of storage time. The number of aldehydes was relatively stable during the storage period but increased after 1 d. Xu et al. also found that the number of aldehydes increased after 1 d storage [29]. During the period, the types of esters and other substances decreased with the extension of storage time. Compared to the decrease in species composition observed in the control group, the ultrasound group showed an increase in the abundance of terpenes and alcohols, which play a key role in species composition.

### 3.2. Volatile Organic Compounds Analysis of Strawberries during Storage

**Figure 2:**
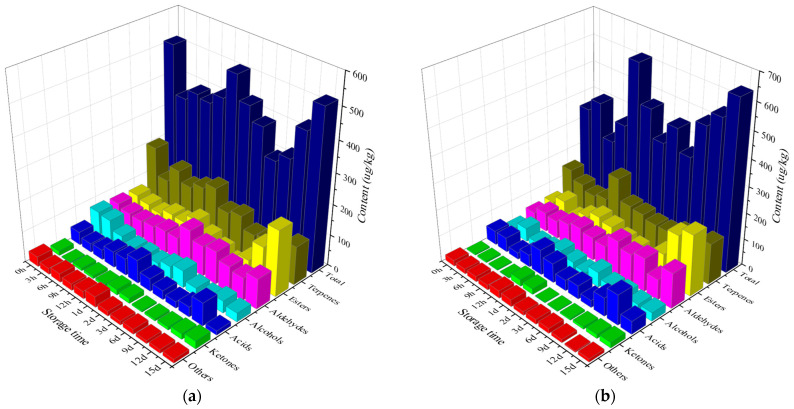
Content of volatile organic components in strawberry fruit during storage. (**a**) Control group; (**b**) ultrasonic group.

As shown in Figure 2 and Table A1, ultrasonic treatment increased the VOC contents during storage. The volatile compounds in the control group had little change after storage for 15 d compared that of storage for 0 h, which was consistent with the findings of Liu et al. [30]. The volatile compounds in the ultrasonic group showed a greater change, increasing from 356.79 μg/kg to 639.40 μg/kg. The total content of volatile compounds in the control group was higher after 9 h of storage, and the contents of VOCs in the ultrasonic group were higher than that in the control group at 12 h and the time points after 3 d. The total contents of VOCs were decreased by ultrasound at 0 h of storage, and increased by ultrasound at 12 h and after 3 d of storage. Similar to the results of hot water dipping treatment, it was found that the stress initially inhibits the metabolism and reduces the content of some substances. However, in this study, the metabolism was activated with increased storage time, increasing the content of VOCs in the treatment group [45].

For various compounds, terpenoids accounted for a relatively high proportion of the total volatile compounds in the first 12 h of storage, among which the linalool content was the highest, reaching peaks around 12 h, followed by menthol and alpinene contents. Among the alcohols, hexanol and other C6 alcohols had higher contents. A similar content relationship was also found by Zhang et al. [20] and Xu et al. [48]. The contents of esters and aldehydes in both groups increased with the extension of storage time, which was consistent with the findings of Macario et al. [49]. Esters and aldehydes in the two groups increased with the extension of storage time. Joseana et al. and Liu et al. proved that UV-C irradiation and IPL treatment could increase the content of esters and aldehydes during storage [30,50]. Ethyl cinnamate, ethyl caproate, and methyl caproate accounted for most of the esters, and the levels of hexanal, 2-hexenal, and nonanaldehyde were higher among the aldehydes. Previous studies have shown that esters could enhance the fruity flavor of fresh strawberries, and esters and aldehydes contribute to the freshness of strawberries [3]. The higher levels of esters and aldehydes in the ultrasonic group at the end of 15 days of storage might be attributed to the fact that ultrasonic treatment could maintain the freshness of strawberries [21].

Therefore, it was speculated that ultrasonic treatment inhibited the metabolism of VOCs, and the content of VOCs was reduced under ultrasonic stress. However, after 12 h, ultrasonic treatment promoted the metabolism of esters and terpenoids, increased the contents of total volatile compounds, followed by an increase in the contents of linalool, hexanol, ethyl ester, hexaldehyde, terpenoids, alcohols, esters, and aldehydes.

### 3.3. Analysis of Key Volatile Compounds in Strawberry

**Figure 3:**
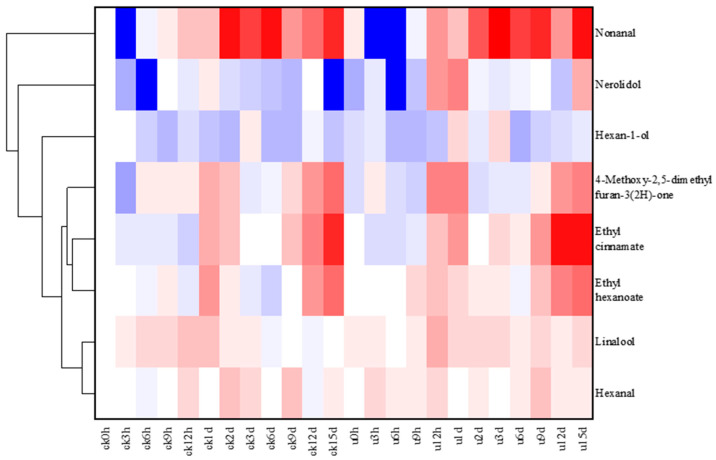
Heatmap of key volatile compounds of strawberry from control and ultrasonic groups. Red color represents the higher expression level of the substances in the sample compared to the sample of ck0h, and blue represents the lower expression level.

Additionally, ultrasonic treatment increased the content of key aroma compounds and improved the fruity aroma of strawberries after 1 d of storage. The aroma activity value OAV (concentration/threshold value) can roughly represent the degree of influence of volatile compounds on the overall aroma of the sample. Compounds with OAV value greater than 1 contribute more to the aroma of the sample. In this study, eight key substances with OAV values greater than 1 were screened (Figure 3 and Table 2), including 1-hexanol, ethyl cinnamate, ethyl caproate, hexanal, nonanaldehyde, linalool, nerolidol, and 4-methoxy-2,5-dimethyl furanone. These compounds were identified as the key contributors to the aroma of the sample, implying that these substances play a pivotal role in the aroma of strawberries [51].

Among the key aroma substances, 4-methoxy-2,5-dimethylfuranone could affect the specific flavor of strawberries [52]. Strawberries continue to synthesize furanones even after the plant reaches full maturity, and the content of furanones increases after harvest [53]. A similar trend was observed in this study. In addition to their significant floral properties [54], terpenoids such as linalool have a series of physiological functions, especially their involvement in plant stress resistance [55]. It is reported that two key ester compounds, ethyl caproate and ethyl cinnamate, which increase with storage time, can impart a fruity and wine-like flavor [28,56]. Aldehydes are associated with the improvement of plant flavor, while hexanal has a grassy flavor. Du et al. also proved that hexanal is one of the characteristic aroma substances of strawberries [21]. Additionally, it is reported that hexanal has the potential to suppress postharvest diseases [57]. Li et al. showed that octanal and nonanal have the antifungal properties [58].

Compared with the control group, the content of 4-methoxy-2,5-dimethylfuranone in the ultrasonic group was lower than that in the control group at 0 h, and the concentration of 4-methoxy-2,5-dimethylfuranone was significantly increased at 12 h and 1 d after ultrasonic treatment. The levels of linalool and nerolidol were higher in the ultrasonic group at most times. The contents of key terpenoids increased gradually after 6 h, with the largest increase at 12 h, 1 d, 12 d, and 15 d. The ultrasonic group showed higher levels of these compounds at 12 h, 1 d, and 15 d. As shown in the accompanying table, ultrasonic treatment significantly increased the hexanol content at 1 d and 3 d. Similar to other substance treatments, ultrasonic treatment increased the contents of aldehydes, especially hexanal and nonanal in the two groups at 12 h, 1 d, 12 d, and 15 d.

These results showed that the contents of ethyl caproate, ethyl cinnamate, and 4-methoxy-2,5-dimethylfuranone were increased at 12 h, 1 d, 12 d, and 15 d compared with those at 0 h, indicating that the fruity flavor of strawberries was more intense after 12 h of storage. Similar to the results of other preservation treatments such as nano-selenium and high-CO_2_ atmosphere [59,60], ultrasonic treatment increased the content of most key flavor compounds at 12 h, 1 d, 12 d, and 15 d.

### 3.4. Metabolic Pathway Analysis

**Figure 4:**
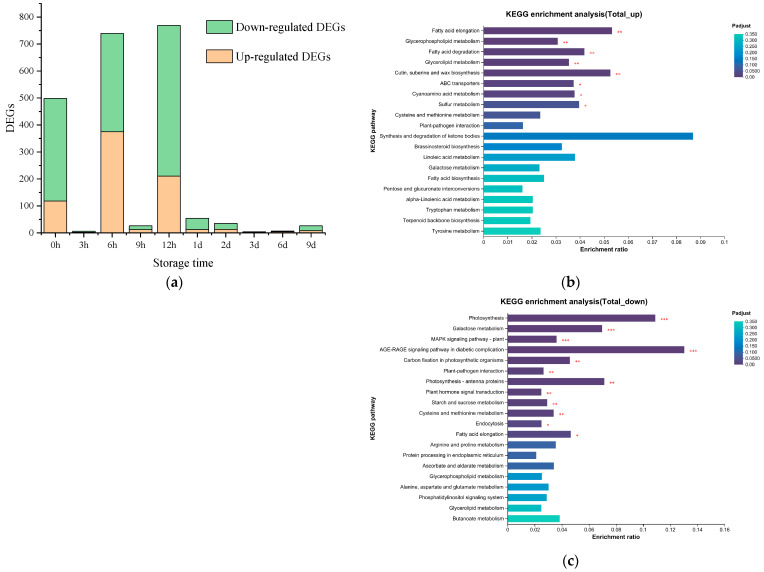
Enrichment analysis of differentially expressed genes of strawberry after treatments. (**a**) Numbers of DEGs in KEGG pathway enrichment analysis of DEGs (|log_2_FC| > 1, *p*-adjust < 0.05) in strawberry fruit after ultrasound treatment. (**b**) Functional enrichment analysis of up-regulated DEGs. (**c**) Functional enrichment analysis of down-regulated DEGs. The column color gradient indicates the significance of enrichment, where Pvalue or Padjust < 0.001 is labeled ***, Pvalue or Padjust < 0.01 is labeled **, and Pvalue or Padjust < 0.05 is labeled *.

The metabolic pathways of aroma substances such as linoleic acid, linolenic acid, and terpenes were activated by ultrasonic treatment, which enhanced the fruity aroma of strawberries during storage. Some studies have found that acoustic signals can induce changes in gene expression [61]. Sound stimulation can help plants defend against other abiotic (drought) or biological (insect) stresses [62]. Ultrasonic treatment can induce the antioxidant system, mainly in the early treatment response expression [63], and improve the fatty acid metabolism pathway. The changes in the key metabolic pathways were analyzed using transcriptome sequencing to explore the molecular mechanism of ultrasonic influence on strawberry aroma metabolism further. The transcriptome sequencing results showed that ultrasonic treatment could significantly affect the gene expression of strawberries. A total of 1905 genes were significantly differentially expressed at different times. In different storage time points, there were more differential genes at 0 h, 6 h, and 12 h (Figure 4a). The transcriptome mainly responds to ultrasonic treatment at the early stage of storage, and the response is mainly down-regulated. Further enrichment analysis of the metabolic pathways showed that the DEGs were more significant in the metabolic pathways such as plant–pathogen interaction and hormone response.

Previous studies have found that ultrasonic treatment could affect plant softening, plant hormone signal transduction, and other metabolic pathways. At the physiological level, ultrasonic treatment can alleviate the softening of strawberry fruits during storage and improve storage quality [7].

Concurrently, differential gene enrichment was observed in the linolenic acid and linoleic acid metabolic pathway, terpenoid skeleton metabolic pathway, and other pathways which are related to ester and terpenoid metabolism of key aroma substances of strawberry [64]. The KEGG enrichment results demonstrated that differential genes were predominantly up-regulated in these pathways, which was analogous to the impact of certain preservation techniques on the transcriptome. Some studies have found that pretreatment before storage can affect the transcriptome expression of fruits; for example, low-temperature storage can enrich DEGs in nectarine in the carbohydrate and terpenoid metabolic pathways [65]. Additionally, cold plasma can regulate the transcriptome of blueberry, and the enrichment pathway is related to the secondary metabolites [63].

### 3.5. Analysis of Key Gene Transcript Expression

**Figure 5:**
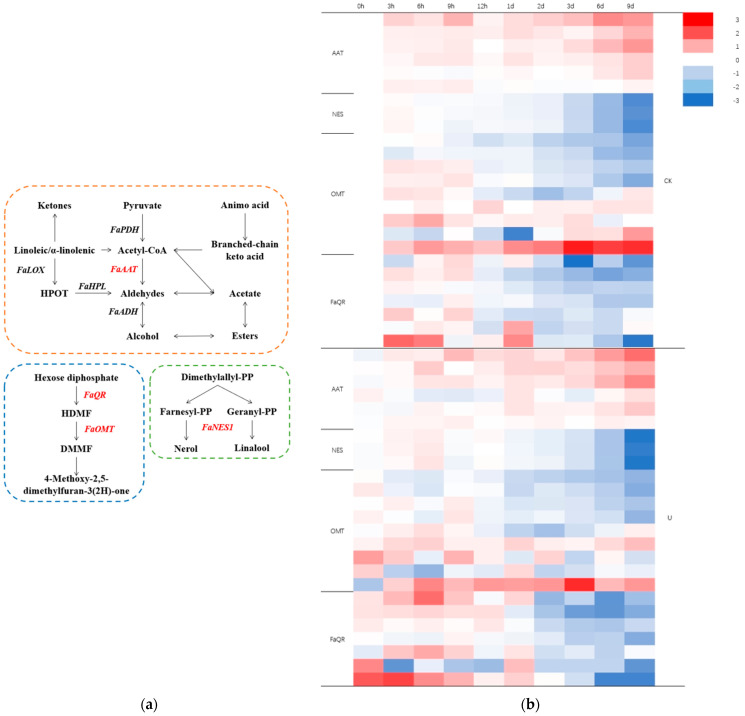
(**a**) Schematic diagram of the synthesis mechanism of strawberry fruit volatile compounds. (**b**) The relative expression of gene transcripts related to the synthesis of volatile compounds in strawberry fruit from control and ultrasonic treatment.

Ultrasound treatment up-regulated the transcription expression levels of genes related to aroma metabolism at some time points. Esters, aldehydes, acids, and alcohols are formed mainly through the metabolism of fatty acids, amino acids and sugars [66,67]. In the fatty acid pathway, fatty acids continuously remove the C2 unit (acetyl-CoA) by β-oxidation to form different fatty acyl-CoA and finally convert to esters (Figure 5). In the amino acid pathway, aliphatic amino acids form branched-chain ketoacids through aminotransferase and then form acyl-CoA through dehydrogenase [68]. Acetyl-coenzyme A is catalyzed to produce acetic acid ester by alkyl transferase AAT or ethanol under the catalysis of reductase and then ethyl acid. Acetyl-CoA is involved in many metabolic pathways and is a common precursor of alcohols, acids, aldehydes, and esters [69]. The activation of the secondary metabolite pathway and an increase in the AAT transcription level to promote the increase in ester aroma were also observed in some genetically mutated mangoes [70].

In the terpenoid metabolic pathway, the nerolidol synthetase (NES) gene *FaNES1* is a key gene encoding the unique “flower fruit” substances of strawberry, while linalool and nerolidol can catalyze the production of linalool and nerolidol by Geranyl-PP or Farnesyl-PP [71]. In a previous study, the levels of NES transcriptional genes were regulated through pollination to regulate the content of terpenes [72]. The carbohydrate pathway mainly produces furanone compounds, representative products like 4-hydroxy-2,5-dimethyl-3(2H)-furanone (HDMF), and 4-methoxy-2,5-dimethyl-3(2H)-furanone (DMMF). FaQR has been shown to be involved in the final step in the synthesis of HDMF from 4-5-methyl-2-methylene-3 (2H) methanone [20]. HDMF is a methylation substrate catalyzed by OMT to produce a more stable DMMF during maturation [73].

The expression of many *FaAAT* transcripts in the ultrasound group was down-regulated compared with that in the control group at 9 h of storage, while some of the transcripts in the ultrasound group were up-regulated after 1 d. Some studies have found that jasmonate can induce the up-regulation of *FaAAT* transcripts in apples [74], and 1-MCP treatment can inhibit the expression level of *FaAAT* in peaches and bananas during storage [75,76], thus affecting the contents of ester substances. Compared with the control group, the expression of *FaNES1* in the ultrasound group was up-regulated at 3 h and 6 h, and no significant difference was observed between the two groups at other time points during storage. The expression of some *FaQR* transcripts in the ultrasound group was more up-regulated than that in the control group within 9 h, then more down-regulated than that in the control group after 1 d. The expression of *FaOMT* transcripts in the ultrasound group was partly up-regulated and partly down-regulated compared with that in the control group at 0 h, and the expression of *FaOMT* transcripts in the ultrasound group was up-regulated compared with that in the control group at 1 d of storage.

### 3.6. Analysis of Real-Time Quantitative Fluorescence 

**Figure 6:**
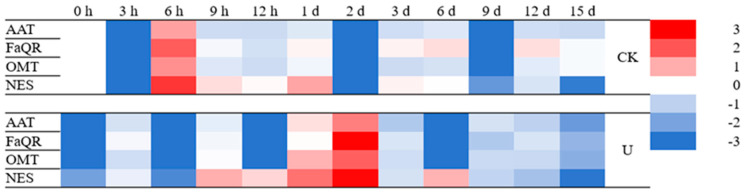
The relative expression of genes related to volatile synthesis of strawberry fruits from the control and ultrasound treatment.

The data obtained by real-time fluorescence quantitative PCR was used to plot a calorimetric map of strawberry aroma gene expression, as shown in Figure 6. The gene expression of aroma metabolism was down-regulated at 0 h and up-regulated at 1 d after ultrasonic treatment. In the control group, the change was significant within 6 h after storage, and the expression level of related genes decreased significantly at 3 h, increased significantly at 6 h, and then decreased significantly at 2 d and 9 d of storage. According to Zhang et al., refrigeration could induce gene expression fluctuations [77]. Compared with the control group, gene expression in the ultrasound group was significantly down-regulated at 0 h and 6 h, which was closely related to the lower substance content in the ultrasound group at 9 h before storage. Gene expression in the ultrasonic group was significantly increased at 1 d and 2 d, and the metabolism of esters, terpenes, and furanones was accelerated, leading to an increase in the volatile compound contents in the ultrasound group after 1 d. The expression of *FaNES* was up-regulated within 6 h–2 d, and the biosynthesis of terpenes was accelerated, which was consistent with the trend of higher content of terpenes around 1 d. After 3–15 d of storage, the expression of the *FaAAT* gene was down-regulated compared with that of the control group at 0 h, but the content of esters still increased with the extension of storage time, indicating that ester catabolism was inhibited. Furthermore, other genes, such as *LOX* and *SAAT*, are also involved in the metabolism of ester substances [28]. Additionally, the enzyme activity of AAT is influenced by the transcription factors, including the MYB group [78]. These factors may influence the content of ester substances, which can be investigated in subsequent experiments.

## 4. Conclusions

In conclusion, this study offers insights into the effects of ultrasound on VOCs and key genes during the storage of strawberries. The results of this experiment showed that the types and contents of VOCs in the ultrasound group decreased at 0 h of storage. However, the contents of total VOCs, especially esters and terpenes, were increased by ultrasonic treatment at 1 d, 12 d, and 15 d of storage. According to the OAV value, eight key aroma compounds were selected, including 1-hexanol, ethyl cinnamate, ethyl caproate, hexanal, nonanaldehyde, linalool, nerolidol, and 4-methoxy-2,5-dimethyl furanone. The content of key aroma compounds such as linalool and ethyl caproate was increased by ultrasonic treatment after 1 d. The results of the transcriptome analysis showed that the metabolic pathways of fatty acids and terpenes were activated by ultrasonic treatment, and the expression levels of *FaAAT*, *FaNES1*, *FaQR,* and *FaOMT* in the ultrasonic group were decreased at 0 h of storage and then increased at 9 h and 1 d of storage, which was consistent with the changes in aroma substances. Therefore, ultrasound could activate the aroma metabolic pathway, improve gene expression, and alleviate the reduction in volatile compound content and variety.

## Figures and Tables

**Table 1:** Primers used for real-time fluorescence quantitative PCR.

Gene Name	Sequences	
*FaAAT*	F	GGGAGGACATCATGGATTG
	R	CTAGATTCACCCACGCTTC
*FaOMT*	F	CACCAGACACTAGCCTTGCC
	R	GGAATCCAGAACCCTTAGCC
*FaQR*	F	AACAATAGTAGGTCCAGCAA
	R	ACATACAAGGGAAAGGAAA
*FaNES*	F	TGGGTCGTATGTAAGGTGC
	R	TGAATGATGCTGGAAATGG

**Table 2:** OAV value of 8 key aroma substance in strawberries.

Name	Threshold	Storage Time											
**Control Group**		**0 h**	**3 h**	**6 h**	**9 h**	**12 h**	**1 d**	**2 d**	**3 d**	**6 d**	**9 d**	**12 d**	**15 d**
Hexanal	5	4.12	3.91	2.99	3.54	5.69	3.98	6.39	5.41	3.61	6.87	3.34	4.30
Ethyl hexanoate	5	5.13	4.31	4.19	5.80	2.79	13.05	6.11	3.05	1.98	4.88	13.89	18.59
Hexan-1-ol	5.6	1.80	1.65	0.75	0.46	0.84	0.62	0.42	2.22	0.43	0.51	1.29	0.53
Nonanal	8	0.54	0.00	0.38	0.63	0.85	0.95	4.88	3.06	4.75	1.38	2.19	4.41
Linalool	6	11.97	13.26	15.46	17.76	21.39	19.81	12.80	14.75	9.79	11.91	8.15	11.18
4-Methoxy-2,5-dimethyl furan-3(2H)-one	16	0.41	0.08	0.45	0.50	0.51	0.94	0.69	0.24	0.32	0.57	1.15	1.59
Nerolidol	10	0.67	0.15	0.00	0.59	0.39	0.79	0.29	0.25	0.24	0.16	0.67	0.00
Ethyl cinnamate	17	0.66	0.41	0.44	0.38	0.27	1.42	1.13	0.58	0.60	1.02	2.31	5.42
**Ultrasonic Group**		**0 h**	**3 h**	**6 h**	**9 h**	**12 h**	**1 d**	**2 d**	**3 d**	**6 d**	**9 d**	**12 d**	**15 d**
Hexanal	5	3.40	5.60	4.48	4.69	6.27	3.50	4.64	3.76	4.46	7.21	4.73	4.63
Ethyl hexanoate	5	5.00	4.84	4.75	7.84	8.47	7.40	5.91	5.72	3.70	8.25	16.57	20.74
Hexan-1-ol	5.6	0.80	1.17	0.48	0.50	0.62	2.62	1.04	2.27	0.37	0.75	0.80	1.11
Nonanal	8	0.55	0.00	0.00	0.40	1.46	0.93	2.75	6.67	3.25	4.33	1.30	5.20
Linalool	6	13.55	13.76	10.62	14.56	23.92	16.35	15.90	15.21	14.77	15.22	13.20	15.23
4-Methoxy-2,5-dimethyl furan-3(2H)-one	16	0.18	0.42	0.18	0.17	1.32	1.39	0.18	0.24	0.23	0.49	1.00	1.18
Nerolidol	10	0.15	0.41	0.00	0.20	1.92	2.03	0.47	0.44	0.47	0.60	0.23	1.49
Ethyl cinnamate	17	0.57	0.33	0.35	0.42	1.09	1.60	0.64	0.89	0.81	1.86	6.02	6.28

## Data Availability

The original contributions presented in the study are included in the article; further inquiries can be directed to the corresponding author.

## Appendix A

**Table A1:** Volatile compounds in strawberries.

Compounds	RT	CAS	Content (μg/kg)												
			Storage Time	0 h	3 h	6 h	9 h	12 h	1 d	2 d	3 d	6 d	9 d	12 d	15 d
Esters															
Ethyl butyrate	7.545	105-54-4	ck	ND	ND	ND	ND	ND	ND	ND	ND	ND	ND	ND	ND
			u	ND	20.22 ± 2.85	ND	ND	ND	ND	ND	ND	ND	ND	ND	ND
Methyl hexoate	11.254	106-70-7	ck	17.25 ± 2.42	19.98 ± 3.24	19.21 ± 2.35	34.56 ± 4.21	50.47 ± 5.35	7.80 ± 1.84	19.50 ± 2.56	25.40 ± 4.21	9.88 ± 1.74	24.45 ± 2.45	27.63 ± 3.62	30.22 ± 5.32
			u	10.86 ± 2.01	28.43 ± 4.25	9.83 ± 2.10	27.37 ± 3.42	34.19 ± 2.46	24.25 ± 4.72	28.92 ± 3.62	18.86 ± 3.23	10.06 ± 1.72	26.50 ± 3.32	16.51 ± 2.32	22.73 ± 3.26
Ethyl hexanoate	12.773	123-66-0	ck	25.63 ± 4.23	21.55 ± 3.23	20.96 ± 3.12	29.00 ± 2.64	13.94 ± 1.24	65.27 ± 8.34	30.53 ± 5.63	15.26 ± 2.34	9.91 ± 1.64	24.40 ± 3.52	69.45 ± 7.35	92.97 ± 10.52
			u	25.00 ± 2.62	24.19 ± 2.73	23.74 ± 3.26	39.21 ± 5.25	42.37 ± 6.74	37.02 ± 4.26	29.55 ± 4.72	28.58 ± 3.72	18.52 ± 2.46	41.27 ± 6.34	82.86 ± 11.46	103.70 ± 12.63
Hexyl acetate	14.181	142-92-7	ck	3.98 ± 1.52	ND	ND	ND	ND	ND	ND	ND	ND	ND	ND	ND
			u	ND	ND	ND	ND	ND	ND	ND	ND	ND	ND	ND	ND
Trans-2-hexenyl acetate	16.638	2497-18-9	ck	9.09 ± 1.26	8.01 ± 2.01	ND	ND	ND	ND	ND	ND	ND	ND	ND	ND
			u	6.13 ± 2.13	ND	ND	ND	ND	ND	ND	ND	ND	ND	ND	ND
Cyclohexyl acetate	16.883	622-45-7	ck	ND	2.54 ± 0.23	ND	ND	ND	ND	ND	ND	ND	ND	ND	ND
			u	ND	2.38 ± 0.54	ND	ND	ND	ND	ND	ND	ND	ND	ND	ND
Methyl benzoate	30.819	93-58-3	ck	ND	ND	ND	0.58 ± 0.12	0.30 ± 0.06	ND	ND	ND	ND	ND	ND	ND
			u	ND	ND	0.87 ± 0.13	0.47 ± 0.10	ND	ND	ND	ND	ND	ND	ND	ND
Gamma-butyrolactone	31.299	96-48-0	ck	0.55 ± 0.08	ND	ND	ND	ND	ND	ND	ND	ND	ND	ND	ND
			u	ND	ND	ND	ND	ND	ND	ND	ND	ND	ND	ND	ND
Methyl salicylate	38.742	119-36-8	ck	ND	ND	ND	0.42 ± 0.14	ND	ND	ND	ND	ND	ND	ND	ND
			u	ND	ND	ND	ND	ND	ND	ND	ND	ND	ND	ND	ND
Methyl cinnamate	51.77	1754-62-7	ck	0.55 ± 0.11	ND	0.24 ± 0.04	0.29 ± 0.06	0.50 ± 0.14	ND	0.30 ± 0.06	0.49 ± 0.10	ND	0.64 ± 0.13	ND	ND
			u	ND	0.29 ± 0.05	ND	0.33 ± 0.04	0.70 ± 0.06	0.64 ± 0.15	ND	0.69 ± 0.21	0.47 ± 0.12	0.84 ± 0.25	ND	ND
Ethyl cinnamate	53.123	103-36-6	ck	11.22 ± 2.61	6.90 ± 1.26	7.42 ± 2.73	6.48 ± 2.71	4.61 ± 1.73	24.09 ± 4.71	19.16 ± 3.72	9.91 ± 2.26	10.19 ± 2.71	17.39 ± 1.28	39.33 ± 4.72	92.22 ± 10.27
			u	9.72 ± 1.83	5.63 ± 0.83	5.91 ± 1.61	7.18 ± 2.31	18.45 ± 3.72	27.24 ± 3.17	10.85 ± 2.72	15.21 ± 2.27	13.84 ± 2.21	31.61 ± 5.62	102.39 ± 17.36	106.79 ± 10.62
Gamma-Decanolactone	58.882	706-14-9	ck	ND	ND	ND	ND	ND	1.62 ± 0.15	ND	ND	ND	ND	ND	ND
			u	ND	ND	ND	ND	ND	ND	ND	ND	ND	ND	ND	ND
Diisobutyl phthalate	61.859	84-69-5	ck	ND	ND	3.05 ± 0.26	ND	ND	ND	ND	ND	ND	ND	ND	ND
			u	ND	ND	ND	ND	4.65 ± 0.45	ND	ND	ND	ND	ND	ND	ND
**Alcohols**															
Isoamylol	13.529	123-51-3	ck	6.41 ± 1.12	ND	7.84 ± 0.92	5.19 ± 0.63	4.29 ± 0.63	3.98 ± 0.42	5.23 ± 0.47	5.26 ± 1.02	ND	ND	3.75 ± 0.51	ND
			u	ND	ND	ND	4.85 ± 0.52	6.16 ± 0.72	ND	3.92 ± 0.42	3.97 ± 0.23	5.22 ± 0.41	4.80 ± 0.85	3.46 ± 0.45	ND
Pentanol	13.562	71-41-0	ck	7.77 ± 1.93	4.61 ± 0.32	9.76 ± 0.89	5.00 ± 0.46	4.43 ± 0.52	4.06 ± 0.35	4.70 ± 0.32	5.80 ± 0.56	ND	5.02 ± 0.67	3.95 ± 0.36	5.96 ± 0.63
			u	3.48 ± 0.43	7.36 ± 1.25	9.38 ± 1.56	ND	5.82 ± 1.31	2.16 ± 0.23	3.27 ± 0.34	3.89 ± 0.25	5.43 ± 0.43	5.00 ± 0.46	3.86 ± 0.25	3.61 ± 0.15
Cis-2-penten-1-ol	16.244	1576-95-0	ck	3.45 ± 0.35	2.72 ± 0.23	ND	3.60 ± 0.51	ND	ND	ND	ND	ND	ND	ND	ND
			u	2.02 ± 0.23	3.55 ± 0.42	ND	3.49 ± 0.14	4.12 ± 0.23	1.50 ± 0.06	ND	ND	ND	ND	2.55 ± 0.24	ND
Hexan-1-ol	17.703	111-27-3	ck	10.07 ± 1.45	9.25 ± 0.82	4.22 ± 0.24	2.56 ± 0.34	4.71 ± 0.45	3.49 ± 0.24	2.35 ± 0.15	12.45 ± 1.26	2.39 ± 0.42	2.85 ± 0.25	7.23 ± 0.92	2.95 ± 0.35
			u	4.48 ± 0.25	6.53 ± 0.45	2.66 ± 0.27	2.81 ± 0.29	3.48 ± 0.49	14.68 ± 1.52	5.83 ± 0.51	12.68 ± 1.53	2.06 ± 0.26	4.20 ± 0.42	4.49 ± 0.64	6.24 ± 0.52
Trans-2-Hexen-1-ol	20.141	928-95-0	ck	11.50 ± 1.64	19.30 ± 2.64	5.01 ± 0.56	ND	7.12 ± 0.62	2.46 ± 0.43	ND	2.13 ± 0.25	1.78 ± 0.15	2.52 ± 0.26	6.37 ± 0.53	1.09 ± 0.15
			u	8.83 ± 1.83	11.65 ± 0.92	ND	5.18 ± 0.63	5.10 ± 0.52	7.76 ± 0.61	1.49 ± 0.11	3.93 ± 0.46	1.56 ± 0.15	ND	ND	1.92 ± 0.29
Cyclohexanol	20.15	108-93-0	ck	15.98 ± 1.51	19.75 ± 2.31	ND	ND	ND	ND	ND	ND	ND	ND	ND	ND
			u	ND	ND	ND	ND	ND	ND	ND	ND	ND	ND	ND	ND
Cis-2-Hexen-1-ol	20.2	928-94-9	ck	13.24 ± 1.42	12.05 ± 2.21	3.19 ± 0.41	5.27 ± 0.45	7.26 ± 0.64	2.65 ± 00.25	ND	ND	ND	ND	ND	ND
			u	ND	12.25 ± 0.98	4.62 ± 0.52	4.79 ± 0.43	5.75 ± 1.06	2.19 ± 0.25	ND	ND	ND	3.01 ± 0.32	2.96 ± 0.31	2.41 ± 0.23
Oct-1-en-3-ol	22.344	3391-86-4	ck	ND	ND	5.15 ± 0.62	ND	ND	2.03 ± 0.43	9.48 ± 1.34	9.07 ± 0.82	7.30 ± 0.91	3.67 ± 0.43	4.21 ± 0.32	ND
			u	ND	4.45 ± 0.32	ND	ND	7.59 ± 0.94	ND	ND	12.13 ± 1.23	6.70 ± 0.42	5.72 ± 0.62	3.37 ± 0.31	ND
2-Ethylhexan-1-ol	24.237	104-76-7	ck	5.54 ± 0.41	3.68 ± 0.23	4.48 ± 0.45	2.36 ± 0.25	ND	4.56 ± 0.15	4.46 ± 0.25	5.01 ± 0.62	5.48 ± 0.56	6.58 ± 0.72	8.70 ± 1.02	11.13 ± 1.93
			u	5.13 ± 0.53	2.59 ± 0.20	3.68 ± 0.53	2.32 ± 0.30	5.53 ± 0.53	3.13 ± 0.24	4.10 ± 0.31	2.80 ± 0.45	6.39 ± 0.53	8.34 ± 0.75	4.97 ± 0.34	6.34 ± 0.25
Octan-1-ol	27.958	111-87-5	ck	2.95 ± 0.36	1.70 ± 0.16	3.57 ± 0.46	3.45 ± 0.26	2.27 ± 0.74	4.46 ± 0.53	17.03 ± 1.93	16.55 ± 0.37	8.73 ± 0.39	2.95 ± 0.32	5.31 ± 0.52	8.15 ± 0.89
			u	2.80 ± 0.34	3.62 ± 0.53	2.15 ± 0.32	2.21 ± 0.22	6.82 ± 0.34	3.60 ± 0.52	6.59 ± 0.63	17.78 ± 1.52	7.12 ± 0.82	8.67 ± 0.93	5.00 ± 0.52	11.96 ± 1.83
2-Phenylethanol	45.972	60-12-8	ck	0.65 ± 0.12	ND	ND	0.39 ± 0.07	ND	ND	ND	ND	ND	ND	ND	ND
			u	0.26 ± 0.12	ND	ND	0.41 ± 0.08	ND	ND	ND	ND	2.13 ± 0.24	ND	ND	ND
**Aldehydes**															
Hexanal	8.452	66-25-1	ck	20.61 ± 3.18	19.56 ± 2.41	14.94 ± 2.12	17.72 ± 2.51	28.45 ± 3.52	19.92 ± 1.51	31.94 ± 3.24	27.04 ± 2.51	18.07 ± 1.72	34.33 ± 4.63	16.69 ± 2.52	21.50 ± 2.16
			u	16.99 ± 1.62	27.98 ± 2.32	22.39 ± 1.62	23.47 ± 2.63	31.34 ± 2.35	17.52 ± 2.15	23.20 ± 3.21	18.78 ± 2.61	22.32 ± 2.61	36.06 ± 3.12	23.65 ± 3.11	23.13 ± 2.62
Trans-2-hexenal	12.361	6728-26-3	ck	38.01 ± 4.61	33.23 ± 3.71	32.02 ± 2.46	30.99 ± 2.36	29.47 ± 3.72	45.00 ± 5.27	37.62 ± 3.61	35.72 ± 4.16	38.99 ± 4.26	27.63 ± 3.71	29.19 ± 3.77	29.19 ± 2.18
			u	19.92 ± 2.71	37.18 ± 4.17	37.18 ± 4.81	43.86 ± 4.16	35.94 ± 3.71	52.36 ± 5.16	28.28 ± 3.93	47.43 ± 4.12	44.59 ± 4.61	39.40 ± 3.36	22.70 ± 2.41	52.40 ± 6.27
Octanal	14.249	124-13-0	ck	ND	ND	ND	ND	ND	2.78 ± 0.31	6.70 ± 0.53	5.12 ± 0.66	4.84 ± 0.34	3.14 ± 0.23	3.30 ± 0.52	4.23 ± 0.33
			u	ND	ND	ND	ND	2.53 ± 0.21	1.79 ± 0.43	2.69 ± 0.22	3.80 ± 0.43	7.15 ± 0.56	3.72 ± 0.25	4.34 ± 0.36	3.52 ± 0.25
Nonanal	19.332	124-19-6	ck	4.36 ± 0.47	ND	3.01 ± 0.31	5.05 ± 0.46	6.81 ± 0.67	7.60 ± 0.83	39.02 ± 3.13	24.47 ± 2.41	38.01 ± 3.15	11.04 ± 1.23	17.55 ± 2.41	35.26 ± 3.15
			u	4.40 ± 0.32	ND	ND	3.23 ± 0.12	11.71 ± 2.31	7.43 ± 0.25	21.98 ± 3.51	53.40 ± 6.32	26.02 ± 3.23	34.65 ± 3.71	10.37 ± 2.01	41.62 ± 4.10
Furfural	22.628	98-01-1	ck	ND	0.94 ± 0.15	ND	ND	ND	ND	ND	ND	ND	ND	ND	ND
			u	ND	ND	ND	ND	ND	ND	ND	ND	ND	ND	ND	ND
Benzaldehyde	25.541	100-52-7	ck	9.60 ± 1.20	7.20 ± 1.23	9.19 ± 0.95	11.40 ± 1.21	9.86 ± 0.41	ND	10.94 ± 1.21	7.06 ± 0.57	7.22 ± 0.48	8.15 ± 1.02	7.59 ± 0.96	8.12 ± 0.83
			u	6.20 ± 0.73	8.62 ± 0.93	7.80 ± 0.94	13.04 ± 1.21	14.03 ± 2.56	9.00 ± 1.02	7.56 ± 0.53	10.59 ± 0.93	12.87 ± 2.10	14.71 ± 1.43	17.05 ± 1.20	13.75 ± 1.56
Undecan-4-olide	58.899	104-67-6	ck	ND	ND	ND	ND	ND	ND	ND	ND	ND	ND	ND	ND
			u	ND	ND	ND	ND	1.73 ± 0.53	ND	ND	ND	ND	ND	ND	ND
**Acids**															
Acetic acid	22.313	64-19-7	ck	2.99 ± 0.45	3.02 ± 0.32	3.83 ± 0.43	1.67 ± 0.34	2.98 ± 0.32	3.79 ± 0.44	ND	4.82 ± 0.45	ND	3.02 ± 0.22	5.91 ± 0.67	4.99 ± 0.43
			u	3.16 ± 0.32	3.43 ± 0.31	3.82 ± 0.43	3.83 ± 0.33	3.69 ± 0.43	8.37 ± 0.64	3.03 ± 0.52	ND	4.98 ± 0.64	4.13 ± 0.36	6.35 ± 0.67	3.84 ± 0.19
2-Methylbutyric acid	33.755	116-53-0	ck	ND	ND	ND	8.61 ± 1.38	ND	ND	ND	ND	ND	ND	ND	ND
			u	ND	2.51 ± 0.32	ND	ND	3.07 ± 0.71	ND	ND	ND	ND	ND	ND	ND
Pentanoic acid	37.311	109-52-4	ck	ND	0.25 ± 0.05	ND	ND	ND	ND	ND	ND	ND	ND	ND	ND
			u	ND	ND	ND	ND	ND	ND	ND	ND	ND	ND	ND	ND
Hexanoic acid	42.853	142-62-1	ck	24.59 ± 3.58	13.51 ± 1.42	24.32 ± 2.53	37.84 ± 4.23	49.69 ± 5.00	61.90 ± 5.19	34.64 ± 3.10	22.04 ± 2.84	20.42 ± 2.12	28.78 ± 3.82	64.36 ± 8.32	8.95 ± 1.49
			u	47.00 ± 4.19	43.27 ± 3.92	18.60 ± 2.94	40.18 ± 4.01	83.01 ± 9.91	56.30 ± 4.92	35.83 ± 3.01	46.75 ± 4.92	20.98 ± 2.27	60.94 ± 4.91	92.70 ± 10.93	43.69 ± 4.12
Octanoic acid	51.332	124-07-2	ck	3.34 ± 0.52	4.94 ± 0.23	2.36 ± 0.33	4.34 ± 0.41	2.05 ± 0.22	8.02 ± 0.84	3.38 ± 0.29	1.52 ± 0.19	1.35 ± 0.20	1.38 ± 0.10	4.81 ± 0.93	ND
			u	2.17 ± 0.23	3.01 ± 0.25	1.81 ± 0.21	3.06 ± 0.41	4.86 ± 0.51	9.57 ± 1.39	2.74 ± 0.42	4.96 ± 0.50	1.95 ± 0.29	5.49 ± 0.48	16.70 ± 1.94	2.20 ± 0.39
Nonanoic acid	54.164	112-05-0	ck	5.97 ± 0.39	3.10 ± 0.42	ND	ND	ND	ND	2.50 ± 0.28	1.37 ± 0.15	ND	ND	ND	ND
			u	ND	ND	ND	ND	ND	ND	ND	ND	ND	ND	ND	ND
Benzoic acid	60.014	65-85-0	ck	6.64 ± 1.03	4.46 ± 0.84	2.61 ± 0.42	ND	ND	2.82 ± 0.32	1.61 ± 0.15	ND	ND	ND	ND	ND
			u	2.29 ± 0.32	3.63 ± 0.35	1.81 ± 0.15	ND	2.35 ± 0.34	ND	ND	ND	ND	ND	ND	ND
**Ketones**															
6-Methylhept-5-en-2-one	17.057	110-93-0	ck	ND	ND	1.62 ± 0.30	ND	ND	3.21 ± 0.32	3.08 ± 0.43	ND	ND	ND	ND	ND
			u	ND	ND	ND	ND	1.27 ± 0.20	ND	ND	ND	ND	ND	ND	ND
4-Methoxy-2,5-dimethylfuran-3(2H)-one	29.556	4077-47-8	ck	6.51 ± 1.02	1.23 ± 0.29	7.15 ± 0.85	7.93 ± 0.95	8.13 ± 1.92	15.06 ± 1.72	11.10 ± 2.02	3.88 ± 0.83	5.13 ± 0.31	9.07 ± 1.09	18.34 ± 1.84	25.39 ± 3.92
			u	2.96 ± 0.42	6.77 ± 0.53	2.95 ± 0.32	2.68 ± 0.23	21.14 ± 0.21	22.30 ± 0.24	2.90 ± 0.37	3.78 ± 0.74	3.70 ± 0.46	7.80 ± 0.91	15.94 ± 1.38	18.96 ± 2.73
Acetophenone	32.305	98-86-2	ck	1.82 ± 0.31	1.58 ± 0.15	ND	1.35 ± 0.11	ND	ND	2.63 ± 0.27	1.37 ± 0.25	1.68 ± 0.15	1.21 ± 0.16	ND	ND
			u	1.63 ± 0.20	ND	1.33 ± 0.15	ND	3.70 ± 0.34	ND	ND	ND	2.75 ± 0.25	1.78 ± 0.14	ND	2.46 ± 0.24
1,5-Ditert-butyl-3,3-dimethylbicyclo[3.1.0]hexan-2-one	52.29	19377-95-8	ck	ND	ND	ND	ND	ND	ND	ND	ND	ND	ND	ND	ND
			u	1.03 ± 0.23	ND	ND	ND	ND	ND	ND	ND	ND	ND	ND	ND
Noroxoagarofuran	61.462	5986-25-4	ck	7.01 ± 0.91	ND	ND	ND	ND	ND	ND	ND	ND	ND	ND	ND
			u	ND	ND	ND	ND	ND	ND	ND	ND	ND	ND	ND	ND
**Terpenes**															
Alpha-pinene	10.507	80-56-8	ck	ND	ND	ND	ND	ND	ND	ND	ND	ND	ND	ND	ND
			u	3.08 ± 0.21	ND	ND	ND	ND	ND	ND	ND	ND	ND	ND	ND
Limonene	11.635	138-86-3	ck	3.77 ± 0.42	2.74 ± 0.23	2.23 ± 0.24	2.69 ± 0.41	2.99 ± 0.32	ND	2.70 ± 0.32	2.82 ± 0.44	2.20 ± 0.23	1.52 ± 0.18	1.78 ± 0.19	1.85 ± 0.21
			u	2.32 ± 0.21	1.98 ± 0.21	2.26 ± 0.22	2.17 ± 0.32	3.63 ± 0.34	ND	2.80 ± 0.26	2.38 ± 0.27	2.41 ± 0.21	1.97 ± 0.19	ND	2.85 ± 0.32
Cineole	11.84	470-82-6	ck	0.94 ± 0.19	ND	ND	ND	ND	ND	ND	ND	ND	ND	ND	ND
			u	0.67 ± 0.09	ND	ND	ND	ND	ND	ND	ND	ND	ND	ND	ND
(Z)-linalool oxide	23.287	5989-33-3	ck	4.78 ± 0.45	1.81 ± 0.19	2.04 ± 0.29	3.09 ± 0.30	3.25 ± 0.31	2.80 ± 0.25	2.28 ± 0.24	2.32 ± 0.22	1.59 ± 0.19	1.54 ± 0.23	1.24 ± 0.21	1.41 ± 0.16
			u	2.84 ± 0.23	3.11 ± 0.42	3.07 ± 0.63	2.25 ± 0.32	6.28 ± 1.21	3.02 ± 0.53	2.40 ± 0.25	1.78 ± 0.43	1.52 ± 0.22	2.02 ± 0.31	2.21 ± 0.34	2.30 ± 0.26
Linalool	27.136	78-70-6	ck	71.84 ± 8.39	79.58 ± 9.10	92.75 ± 8.38	106.58 ± 15.28	128.36 ± 10.38	118.83 ± 11.39	76.83 ± 9.38	88.51 ± 7.29	58.72 ± 6.29	71.46 ± 5.29	48.91 ± 5.10	67.09 ± 7.29
			u	81.28 ± 8.39	82.59 ± 8.03	63.70 ± 7.03	87.37 ± 9.38	143.52 ± 10.39	98.11 ± 9.48	95.42 ± 10.37	91.23 ± 9.57	88.64 ± 8.93	91.31 ± 10.39	79.19 ± 8.94	91.41 ± 9.38
Terpinine-4-ol	30.014	562-74-3	ck	24.65 ± 2.93	12.68 ± 1.29	20.57 ± 3.02	6.16 ± 1.20	5.98 ± 0.84	19.19 ± 2.30	12.12 ± 1.20	14.24 ± 1.62	9.18 ± 0.84	9.48 ± 0.72	7.35 ± 0.82	16.40 ± 1.47
			u	20.84 ± 2.48	9.23 ± 1.29	10.78 ± 1.47	3.80 ± 0.48	16.04 ± 0.94	6.37 ± 0.73	9.87 ± 0.92	5.58 ± 0.56	12.79 ± 0.74	14.47 ± 1.37	5.67 ± 0.46	18.68 ± 2.48
Menthol	32.103	89-78-1	ck	66.39 ± 5.83	7.32 ± 0.78	36.04 ± 5.38	6.78 ± 1.36	5.06 ± 0.67	20.06 ± 2.04	14.03 ± 1.37	13.26 ± 0.95	10.61 ± 0.93	7.16 ± 0.45	5.02 ± 0.35	29.99 ± 3.47
			u	21.10 ± 2.02	7.77 ± 0.76	8.77 ± 1.02	8.56 ± 0.84	12.95 ± 1.27	ND	11.12 ± 1.40	5.00 ± 0.67	ND	12.22 ± 1.38	7.24 ± 0.85	15.50 ± 0.92
Terpineol	35.142	98-55-5	ck	10.56 ± 1.28	3.86 ± 0.45	7.14 ± 0.74	ND	2.27 ± 0.32	ND	3.24 ± 0.42	3.59 ± 0.32	3.08 ± 0.31	2.56 ± 0.30	2.95 ± 0.29	5.95 ± 0.65
			u	5.91 ± 0.76	2.76 ± 0.34	3.33 ± 0.32	ND	7.44 ± 0.67	3.83 ± 0.43	ND	2.58 ± 0.35	3.61 ± 0.36	4.30 ± 0.54	5.05 ± 0.58	4.64 ± 0.32
Piperitone	36.546	89-81-6	ck	11.06 ± 1.48	ND	4.20 ± 0.74	ND	ND	4.45 ± 0.43	ND	ND	ND	ND	ND	2.81 ± 0.53
			u	6.14 ± 0.74	ND	ND	ND	4.28 ± 0.32	ND	ND	ND	ND	3.30 ± 0.43	ND	5.34 ± 0.56
Nerolidol	50.741	7212-44-4	ck	6.69 ± 0.78	1.48 ± 0.32	ND	5.89 ± 1.02	3.95 ± 0.43	7.89 ± 0.84	2.94 ± 0.32	2.51 ± 0.32	2.35 ± 0.21	1.62 ± 0.18	6.69 ± 0.78	ND
			u	1.47 ± 0.17	4.12 ± 0.43	ND	2.04 ± 0.23	19.19 ± 2.01	20.30 ± 2.93	4.73 ± 0.43	4.44 ± 0.32	4.72 ± 0.56	6.02 ± 0.64	2.29 ± 0.32	14.93 ± 1.42
**Others**															
Toluene	7.575	108-88-3	ck	13.58 ± 1.24	15.54 ± 1.64	17.59 ± 2.83	19.46 ± 2.83	22.79 ± 2.735	23.04 ± 2.65	11.32 ± 1.27	9.55 ± 0.95	8.23 ± 0.75	7.49 ± 0.74	10.86 ± 0.91	8.26 ± 1.38
			u	15.85 ± 1.52	15.74 ± 1.38	15.84 ± 1.73	16.45 ± 1.82	20.57 ± 2.73	18.13 ± 2.10	11.73 ± 1.02	16.46 ± 1.28	10.77 ± 0.94	8.86 ± 0.83	ND	8.67 ± 0.93
Ethylbenzene	9.949	100-41-4	ck	2.90 ± 0.34	ND	ND	ND	ND	ND	ND	ND	ND	ND	ND	ND
			u	ND	ND	ND	ND	ND	ND	ND	ND	ND	ND	ND	ND
Styrene	13.093	100-42-5	ck	ND	ND	1.53 ± 0.20	ND	ND	ND	ND	ND	ND	ND	ND	ND
			u	ND	ND	ND	ND	ND	ND	ND	ND	ND	ND	1.16 ± 0.23	ND
M-cymene	13.318	535-77-3	ck	ND	ND	ND	ND	ND	ND	ND	ND	ND	1.44 ± 0.21	ND	ND
			u	1.55 ± 0.18	ND	ND	ND	ND	ND	ND	ND	ND	ND	ND	ND
P-cymene	14.011	99-87-6	ck	3.44 ± 0.45	2.57 ± 0.32	2.53 ± 0.23	1.92 ± 0.21	1.37 ± 0.19	2.52 ± 0.28	2.39 ± 0.29	1.52 ± 0.20	1.88 ± 0.31	ND	ND	1.70 ± 0.19
			u	ND	2.00 ± 0.29	1.63 ± 0.21	1.03 ± 0.19	1.99 ± 0.17	1.65 ± 0.15	1.92 ± 0.20	1.63 ± 0.18	1.97 ± 0.32	1.67 ± 0.27	2.57 ± 0.31	2.07 ± 0.29
1,3-Dimethyl-5-ethylbenzene	14.052	934-74-7	ck	ND	ND	2.08 ± 0.28	ND	ND	ND	ND	ND	ND	ND	ND	ND
			u	ND	ND	ND	ND	ND	ND	ND	ND	ND	ND	ND	ND
2,5-Dimethylpyrazine	16.858	123-32-0	ck	1.02 ± 0.09	ND	ND	ND	ND	ND	ND	ND	ND	ND	ND	ND
			u	ND	ND	ND	ND	ND	ND	ND	ND	ND	ND	ND	ND
2-(2-Ethoxyethoxy)ethanol	31.527	111-90-0	ck	2.42 ± 0.28	ND	ND	ND	ND	ND	ND	ND	ND	ND	ND	ND
			u	1.21 ± 0.17	ND	1.43 ± 0.18	0.92 ± 0.08	ND	ND	ND	ND	ND	ND	ND	ND
1-Methylnaphthalene	42.353	90-12-0	ck	0.34 ± 0.04	ND	ND	ND	ND	ND	ND	ND	ND	ND	ND	ND
			u	ND	ND	ND	ND	ND	ND	ND	ND	ND	ND	ND	ND
Butylated hydroxytoluene	45.532	128-37-0	ck	1.64 ± 0.26	ND	ND	ND	ND	4.72 ± 0.57	ND	ND	2.54 ± 0.21	ND	ND	ND
			u	ND	ND	ND	ND	3.28 ± 0.32	4.57 ± 0.65	ND	ND	2.79 ± 0.21	ND	ND	ND
Benzothiazole	47.414	95-16-9	ck	1.96 ± 0.19	1.10 ± 0.14	1.31 ± 0.15	0.85 ± 0.11	ND	1.89 ± 0.18	1.03 ± 0.21	1.37 ± 0.13	2.14 ± 0.25	1.12 ± 0.21	1.09 ± 0.10	1.74 ± 0.15
			u	1.33 ± 0.21	0.92 ± 0.09	0.92 ± 0.10	ND	2.05 ± 0.21	1.43 ± 0.24	0.89 ± 0.10	0.91 ± 0.08	2.10 ± 0.28	1.62 ± 0.16	1.39 ± 0.18	1.73 ± 0.13
M-Cresol	49.356	108-39-4	ck	ND	ND	ND	ND	ND	1.68 ± 0.19	ND	ND	ND	ND	ND	ND
			u	ND	ND	ND	ND	ND	ND	ND	ND	ND	ND	ND	ND
Phenol	49.396	108-95-2	ck	1.10 ± 0.19	0.63 ± 0.05	ND	ND	ND	ND	ND	ND	ND	ND	ND	ND
			u	0.57 ± 0.04	ND	0.75 ± 0.10	ND	ND	ND	ND	ND	ND	ND	ND	ND
2,4-Di-t-butylphenol	57.16	96-76-4	ck	4.55 ± 0.56	0.92 ± 0.19	3.88 ± 0.41	ND	0.34 ± 0.05	1.32 ± 0.21	1.52 ± 0.14	0.97 ± 0.19	1.43 ± 0.17	ND	ND	0.87 ± 0.11
			u	1.80 ± 0.21	0.64 ± 0.17	0.53 ± 0.08	0.42 ± 0.05	0.93 ± 0.09	1.27 ± 0.14	ND	ND	1.41 ± 0.17	0.51 ± 0.04	ND	ND

Ck: Control group, U: Ultrasonic group. ND means not detected.
